# Barriers in distribution, ownership and utilization of insecticide-treated mosquito nets among migrant population in Myanmar, 2016: a mixed methods study

**DOI:** 10.1186/s12936-019-2800-4

**Published:** 2019-05-14

**Authors:** Shwe Yi Linn, Thae Maung Maung, Jaya Prasad Tripathy, Hemant Deepak Shewade, Swai Mon Oo, Zaw Linn, Aung Thi

**Affiliations:** 1 Vector Borne Disease Control, Naypyi Taw, Southern Shan State Myanmar; 2 Department of Medical Research, Ministry of Health and Sports, Yangon, Myanmar; 3 International Union Against Tuberculosis and Lung Disease, The Union South East Asia Office, New Delhi, India; 4 International Union Against Tuberculosis and Lung Disease, Paris, France; 5 All India Institute of Medical Sciences, Nagpur, India; 6 Population Services International, Yangon, Myanmar; 7 Vector Borne Disease Control Programme, Ministry of Health and Sports, Naypyi Taw, Myanmar

**Keywords:** Insecticide-treated bed net, Malaria, Myanmar, Structured Operational Research Training IniTiative (SORT IT)

## Abstract

**Background:**

Sleeping under insecticide-treated mosquito nets/long-lasting insecticidal nets (ITNs/LLINs henceforth referred to as ITNs) is one of the core interventions recommended by the World Health Organization to reduce malaria transmission and prevent malaria in high-risk communities, such as migrants, by preventing mosquito bites. The malaria burden among the migrant population is a big challenge for malaria elimination in Myanmar. In this context, this study aimed to assess the ownership and utilization of ITNs and to understand the barriers to distribution and utilization of ITNs among the high-risk migrant communities in the Regional Artemisinin Resistance Initiative (RAI) project areas of Myanmar.

**Methods:**

A sequential mixed methods study (quantitative component: cross-sectional study involving analysis of secondary data available from a survey conducted among migrant households in the RAI project areas of Myanmar in 2016 followed by a descriptive qualitative component in 2018). A total of 17 focus group discussions (involving 121 participants) with different groups of migrants and 17 key-informant interviews with key programme stakeholders were conducted in 4 selected townships of RAI project areas.

**Results:**

Of 3230 migrant households, 63.3% had at least one ITN while 36% had sufficient ITNs (i.e., 1 ITN per 2 persons). Regarding ITN utilization, about 52% of household members reported sleeping under an ITN the previous night, which is similar among under-fives and pregnant women. Over half of all bed nets were ITNs, with nearly one-third having holes or already undergone repairs. The qualitative findings revealed that the key challenges for ITN utilization were insufficient ITNs in households and dislike of ITNs. The barriers to ITN distribution were incomplete migrant mapping due to resource constraints (time, money, manpower) and difficulties in transportation and carrying ITNs.

**Conclusion:**

This study highlights poor ownership and utilization of ITNs among migrants in the RAI project areas of Myanmar and barriers to their ownership and utilization. To achieve universal coverage and utilization, more programmatic support by the programme is needed to carry out complete migrant mapping and continuous ITN distribution in remote locations.

## Background

Despite the progress made, malaria is a significant public health problem. In 2016, an estimated 216 million cases of malaria occurred worldwide claiming 445,000 lives [1]. About 3.2 billion people in 91 countries are at risk of *Plasmodium* infection in the world [1].

Insecticide-treated bednets or long-lasting insecticidal nets (ITNs/LLINs—henceforth referred to as ITNs) are one of the core interventions recommended by the World Health Organization (WHO) to reduce malaria transmission and prevent malaria in high-risk communities by preventing mosquito bites [2]. Bednets have been shown to reduce the incidence of uncomplicated malaria cases by 50%, severe malaria by 45% in a variety of settings and malaria mortality by 55% in children [3].

Myanmar has a high burden of malaria with more than two-thirds of the population at risk [4]. Myanmar tops in terms of contribution of malaria cases among the countries in the Greater Mekong Sub-region (GMS), which is known for artemisinin resistance [5].

Myanmar is committed towards eliminating malaria by 2030. In order to accomplish that, the country aims to achieve and maintain 100% access and utilization of ITNs at the household level. Free distribution of ITNs in areas of high malaria transmission is one of the key interventions for malaria elimination in Myanmar. This is carried out mainly by the National Malaria Control Programme (NMCP) and other partners. The NMCP also plans for continuous ITN distribution especially in high-risk population groups. Nearly 11 million ITNs have been distributed free of cost in the last 5 years [6]. However, there is little information about the actual utilization of ITNs. There were also reports of misuse of ITNs such as use of nets for fishing among migrant plantation workers in Myanmar [7].

Myanmar has a huge migrant population. In 2014, 9.4 million people were internal migrants (20% of the population) [8]. Migrants are vulnerable to poor health access and treatment, often leading to worsening of health outcomes. Malaria burden among the migrant population is a big challenge for malaria elimination in the country. This issue is particularly important for mobile migrants working in remote forested areas in Myanmar and in the GMS who face major barriers in accessing malaria diagnosis and treatment services [9]. The high mobility of this population is one of the main limitations for malaria control and elimination, particularly on the Myanmar-Thai border [9]. This migration also facilitates the spread of artemisinin-resistant parasites across international borders in the region. There is poor surveillance of malaria and poor uptake of preventive and curative services in these groups [4]. The NMCP recommends better targeting of these hard-to-reach populations. A strategic framework for artemisinin resistance in Myanmar (MARC) by the Union of Myanmar and the WHO also outlines improving access to and use of malaria care services by the mobile/migrant population as a key objective of the containment framework [10]. In this context, it is useful to understand the ownership and utilization of ITNs and reasons for its poor utilization among high-risk migrant communities.

There are several studies across the globe reporting the gap between ownership and utilization of ITNs [4, 11]. Few studies from Myanmar have explored this aspect in the general population and specific migrant occupations, such as plantation workers [7, 12, 13]. However, there is limited information among migrant populations in high-risk areas of artemisinin resistance. Also, the reasons for poor utilization of ITNs from users’ perspective and barriers to distribution of ITNs from providers’ perspective has little precedence in literature. Those that exist are mostly from African countries which are quite different settings in terms of their demographics and social characteristics, health infrastructure and malaria epidemiology [14–16]. Few studies have looked at net care and repair behaviour of ITNs without actually exploring the utilization aspect of the nets [17, 18]. The present study was conducted with the following objectives: among the migrant population in the Regional Artemisinin Initiative (RAI) areas of Myanmar, to i) assess the physical condition, ownership and utilization of bed-nets and, ii) explore barriers to distribution and utilization of ITNs.

## Methods

### Study design

A sequential mixed methods study (quantitative component: cross-sectional study involving analysis of secondary data available from a survey conducted among migrant population in the RAI areas of Myanmar in 2016 followed by a descriptive qualitative component). Content analysis was done using a combination of inductive and deductive coding [19].

### Setting

#### Myanmar National Malaria Control Programme

In Myanmar, 291 out of 330 townships are malaria endemic with about 44 million at risk of the disease. In recent years, Myanmar has made significant progress in reducing malaria morbidity and mortality by 65 and 97%, respectively, (2015 vs 2007). The NMCP is committed to eliminate malaria from the entire country by 2030 [4]. Six states/regions: Bago, Magway, Mandalay, Nay Pyi Taw, Mon, and Yangon are in elimination phase, with a malaria incidence of < 1 case per 1000 population/year, while others are still in the transmission reduction phase where malaria incidence is 1 or above case per 1000 population/year [4]. All malaria diagnostic and treatment services are offered free of cost.

#### RAI areas

**Fig. 1:**
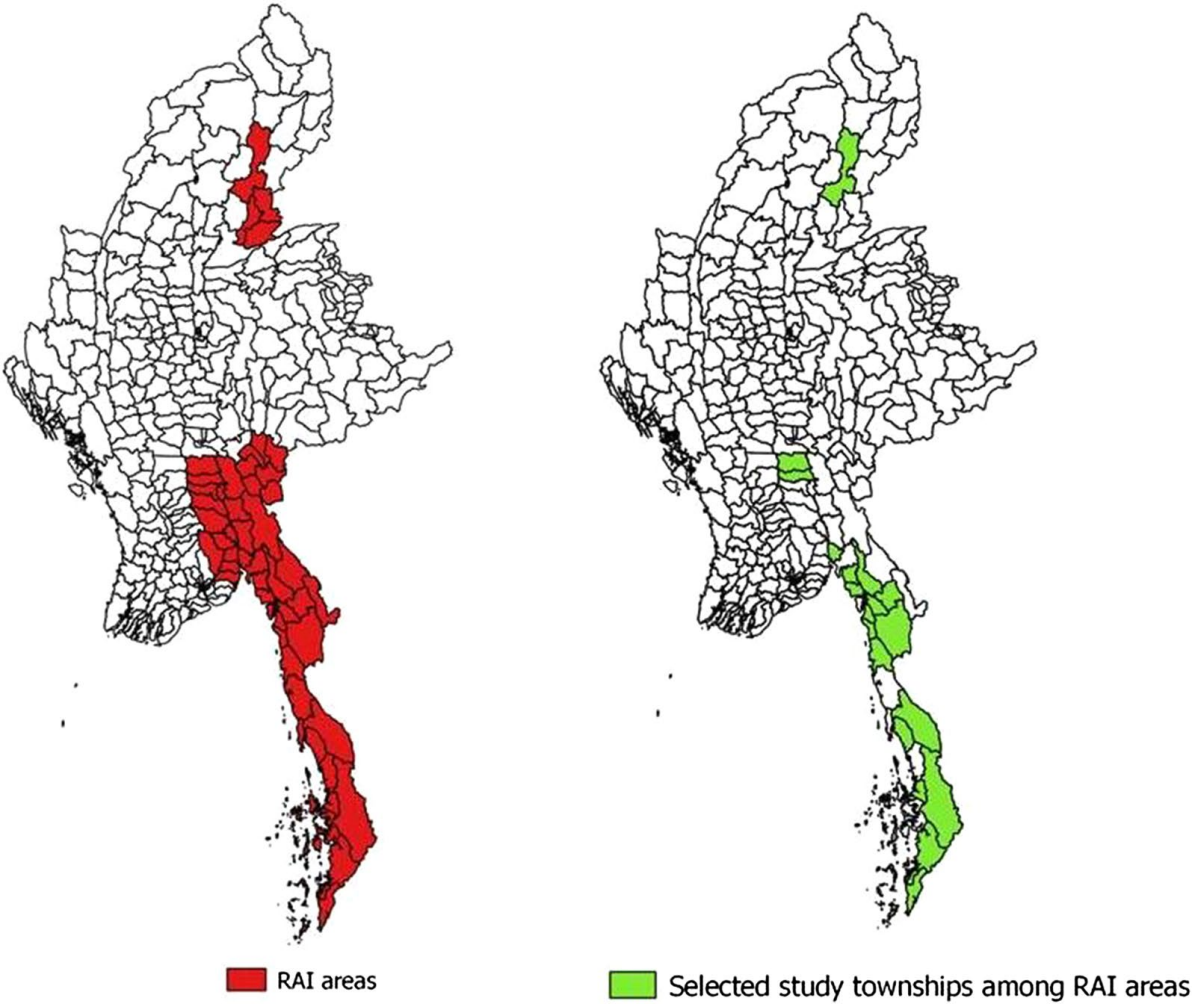
Map showing the Regional Artemisinin-resistance Initiative townships of Myanmar and the Regional Artemisinin-resistance Initiative townships selected for the survey, 2016

Myanmar is one of the countries in the GMS known for artemisinin resistance. Myanmar Artemisinin Resistance Containment (MARC) framework was developed in 2011 to control the emergence of artemisinin resistance. Areas of artemisinin resistance are stratified into three tiers (*tier 1:* areas with credible evidence of artemisinin resistance; *tier 2:* areas with significant inflow of people from tier 1, including those immediately bordering tier 1; *tier 3:* areas with no evidence of artemisinin resistance and limited contact with tier 1). There are 31 townships in tier 1, 21 in tier 2 and 258 in tier 3. In 2013, the project was transferred to the Three Millennium Development Goals Fund (3MDG) and renamed the Regional Artemisinin Resistance Initiative (RAI) in 2014. RAI project areas include 52 townships in tiers 1 and 2 (Fig. 1). The project was gradually expanded to 72 townships in 2015 and 76 in 2016 [4]. The overall goal of the project is to prevent the emergence or spread of artemisinin resistance to new areas [4]. The present study was carried out in selected townships of RAI areas.

#### Distribution of ITNs

Before bed-net distribution, migrant mapping is carried out which lists all household members eligible for ITNs. This is carried out by local vector borne disease control (VBDC) staff, basic health staff (BHS) and village health volunteers (VHVs) under the guidance of the state/regional VBDC Team Leader and Regional Officer (Malaria). This is followed by distribution of nets according to the list prepared.

#### Migrant survey

A community-based survey was conducted jointly by the Department of Medical Research (DMR) and the NMCP in 2016 to understand the knowledge, attitude and health-seeking behaviour towards malaria and the ownership and utilization of ITNs among the migrant population in selected townships of RAI areas. A total of 3230 households in 125 migrant sites located in 27selected townships were covered, out of which all 3230 (100%) respondents completed the interview.

In the selected households, face-to-face interviews were conducted with preferably the female adult respondent or any other adult, using a semi-structured questionnaire by trained interviewers. Questionnaires were pre-tested among the migrants who resided in the sites that were not part of the study in the selected townships. Those in the pre-test were excluded from the study. The questionnaire mostly had close-ended questions with multiple choices and a few open-ended questions without any follow-up questions or further probes.

All the interviewers were trained in each state/region by NMCP staff under the guidance of researchers from the DMR. The local non-health volunteers with high school or higher level of education were trained. Training sessions were performed at the local VBDC office. Training content included seeking informed consent, sampling of study sites and households, interview technique, practical exercise on administering survey questions, role of interviewers and supervisors in data collection, checking, compilation and sending completed questionnaires. The duration of training was 2 days (1 day training followed by practical exercise) which involved interviews with respondents, and then feedback and discussion the next day. Each participant had to perform at least two household interviews.

After data collection, completed questionnaires of each participant were checked immediately by supervisor or another interviewer in the absence of supervisor. Both supervisor and interviewer had to sign the completed questionnaire after checking for accuracy and completeness. Survey data were double entered and validated using EpiData Entry software (version 3.1, EpiData Association, Odense, Denmark). This community-based malaria survey database is available with the NMCP, Ministry of Health and Sports, Myanmar.

#### Data variables and data collection

For the quantitative part, variables related to the bed net, washing of bednet and its utilization such as source, type, size, and physical condition of bednet, soaked in insecticide, number of bednets per household, number of people sleeping under it, frequency of washing, material used for washing, way of drying etc. were extracted from the survey database.

For the qualitative component, barriers in the distribution and utilization of ITNs were explored through key informant interviews (KIIs) with key programme stakeholders and focus group discussions (FGDs) with selected migrant population belonging to various occupational groups.

The principal investigator (PI) along with a team of trained qualitative researchers conducted the KIIs and FGDs on a day, time and place convenient to the participants after obtaining their permission and written informed consent. The KIIs were conducted in the community or the nearby health facility as per the convenience of participants whereas the FGDs were carried out in the community. The PI did not have a prior relationship with any of the participants and was not involved in the direct provision of medical care of the community members. Participants were informed of the purpose of the study and its relevance to the programme prior to the KII/FGD. The interviewer (PI) introduced herself by saying that she is a staff of the NMCP and is doing this research in order to understand the barriers to the distribution and utilization of ITNs among migrant communities and that the study results will be presented to the national programme managers. This will support development of effective strategies to improve ITN utilization which will in turn reduce malaria morbidity and mortality in this vulnerable group. During the KIIs/FGDs, the facilitator was supported by a note-taker. A KII/FGD guide with broad open-ended questions with probes was prepared (Annexure 1). The FGD guide was pilot-tested in similar population in another migrant site not part of the study, whereas the KII guide was administered to other programme stakeholders. The interviews and FGDs were audio recorded after taking consent. On average, the KIIs and FGDs lasted for around 30 min and 45 min, respectively. Verbatims were also noted down by the facilitator during data collection. These are words/statements or in other words quotations exactly spoken by the participant to support the arguments made under the qualitative findings. Field notes (reflections or observations made by the facilitator) were taken during and/or after the interview or FGDs. These notes provided the necessary context and helped recollect the events while going through the transcripts. Definitions of the indicators related to the ownership and utilization of bed nets are given in Box 1.

**Table:** Box 1 Definitions of key terms related to ownership and utilization of bed nets

*Proportion of households with at least one ITN* Numerator: Number of households surveyed with at least one ITN Denominator: Total number of households surveyed *Proportion of households with at least one ITN for every two people* Numerator: Number of households surveyed with at least one ITN for every two people Denominator: Total number of households surveyed *Proportion of the population with access to an ITN in their household* Numerator: Total number of individuals who can sleep under an ITN if each ITN in the household is used by two people Denominator: Total number of individuals who spent the previous night in the surveyed households *Proportion of the population that reported sleeping under an ITN the previous night* Numerator: Number of individuals who reported sleeping under an ITN the previous night Denominator: Total number of individuals who spent the previous night in the surveyed households *Migrant population* Migrant population, according to the survey, was defined as a mobile person of any age who temporarily lives in the selected townships for less than 3 years duration of stay and not registered as a native villager in the village census	

### Study population, sampling and sample size

#### Quantitative component

For the quantitative component, the study population included all migrants residing in the selected townships of RAI areas in 2016. The survey employed a multistage sampling procedure. Out of 52 townships in RAI areas, 13 townships were excluded due to political reasons and being hard-to-reach and conflict areas. From the remaining 39 townships, 27 were selected by probability proportional to size (PPS) (Fig. 1). In each selected township, ~ 5 migrant sites were selected. A total of ~ 125 households were selected from 5 migrant sites in each township. In each site, the households were systematically selected at equal intervals (the interval was determined by the formula: total number of households to be selected/total number of households in the site) from a list of households that was prepared from a migrant mapping exercise done prior to the survey. A total of 3230 households (~ 125 households from each township) were selected. From each household, a respondent, preferably an adult female was selected for interview. These respondents were mostly migrants who came to work or in search of jobs, belonging to various ethnic groups and occupations, such as farming/rubber tapping/stone mining/brick-kiln work/daily wage labour, etc. Most of them stayed in rural remote forest locations. The detailed socio-demographic features of the sample are given in Table 1.

**Table 1:** Socio-demographic characteristics of migrant households in Regional Artemisinin-resistance Initiative areas of Myanmar, 2016

Characteristics	Weighted		Unweighted
	N	%	N
Total number of households	3230		3230
Type of migrant settlement^a^			
Category 1	756	(23.4)	778
Category 2	982	(30.4)	955
Category 3	1251	(38.7)	1248
Missing	242	(7.48)	249
Education of head of household			
No formal education	260	(8.0)	264
Up to primary	2464	(76.3)	2426
Up to secondary	197	(6.1)	213
Up to high school	241	(7.5)	250
Higher education	65	(1.9)	74
Missing	3	(0.2)	3
Occupation of head of household			
Farming/gardening/rubber tapper	1231	(38.1)	1147
Stone mining work/Brick kiln work	817	(25.3)	780
Merchant	38	(1.2)	65
Daily wage labourer	572	(17.7)	610
Missing	572	(17.7)	628
Working hours			
Daytime	2486	(77.0)	2501
Night time	501	(15.5)	479
Missing	243	(7.5)	250
Reason for moving here			
To work	2276	(70.5)	2188
To find job	358	(11.1)	489
To live here	163	(5.1)	179
Living with spouse/relatives	197	(6.1)	196
Others	15	(0.5)	18
Missing	36	(1.1)	22
Intention to stay			
Less than 2 weeks	17	(0.5)	17
2–4 weeks	13	(0.4)	13
1–6 months	385	(11.9)	296
6 months to 1 year	204	(6.3)	185
More than 1 year	366	(11.3)	408
Not sure	1959	(60.6)	2031
Missing	287	(8.9)	280
State/region			
Tanintharyi	876	(27.1)	748
Kayin	401	(12.4)	374
Shan (East)	27	(0.9)	131
Mon	839	(26.0)	875
Kachin	149	(4.6)	247
Sagaing	590	(18.3)	626
Bago	347	(10.8)	229
Number of household members			
1–2	1153	(35.7)	1115
3–5	1623	(50.2)	1655
6–10	446	(13.8)	451
More than 10	08	(0.3)	9

^a^Types of migrant settlement: Category 1 = Company owned large compound; Category 2 = Medium sized compound; Category 3 = Owned small business-gold mining/forest workers; Figures presented here are weighted estimates; figures given in parentheses are percentages

#### Qualitative component

For the qualitative component, a sample of migrant population (belonging to different occupational groups) residing in 4 selected townships of RAI areas were selected to explore the users’ perspective. Village health volunteers, local VBDC staff, BHS and regional medical officer from the same areas as the migrants were interviewed for understanding the providers’ perspectives.

Two state/regions (Bago and Sagaing) in RAI areas, having large sites of migrant population with wide variations in occupational characteristics, were purposively selected. In each state/region, two townships (Taungoo and Yedashe Townships in Bago Region, Homalin and Kalay Townships in Sagaing Region) were selected by convenience sampling.

KIIs were conducted with the Health Assistant (n = 2), Malaria Assistant (n = 2), Malaria Supervisor (n = 3), Regional Medical Officer (n = 1) at state/region level, and VHVs (n = 8) at village level working under the NMCP in selected townships. Participants were selected by convenience sampling.

Seventeen FGDs were also conducted with selected migrant populations belonging to different occupational groups. Each FGD consisted of around 6–10 participants. Purposive sampling was used to recruit these FGD participants. FGD participants in each migrant site were selected to get representation from different occupations and demographic profile (gender and age). The participants belonged to different occupations such as farmer, fishermen, gold miner, forest goer, brick kiln worker, stone mine worker, daily wage labourer etc. The numbers of KIIs/FGDs were based on the saturation of findings. Participants were recruited until no new relevant information pertaining to the three major themes was being obtained. In the FGDs, non-receipt of bednets/insufficient bednets and dislike of bednets were being repeated. In the KIIs, logistic challenges in carrying out migrant mapping and distribution of nets such as transportation, manpower, time and cost featured repeatedly. After/during each interview/FGD, the summary of the findings were shared with the participants for their feedback. In case of any feedback, clarification was sought and consensus was arrived at.

### Data analysis and statistics

#### Quantitative analysis

Data were extracted from the survey database and imported into STATA (version 11, StataCorp, TX, USA) for analysis. Proportions (with 95% confidence intervals) were used to summarize categorical variables related to ownership, utilization and physical condition of bednets. Due to the multistage sampling technique adopted in this survey, sampling weights (inverse of the probability of selection of each respondent) were calculated for each respondent and weighted estimates have been presented. This is likely to increase the generalizability of the study findings to the larger population of interest. Logistic regression was performed to explore the socio-demographic factors associated with household net ownership. Adjusted odds ratio (OR) was used to measure the strength of this association.

#### Qualitative analysis

**Fig. 2:**
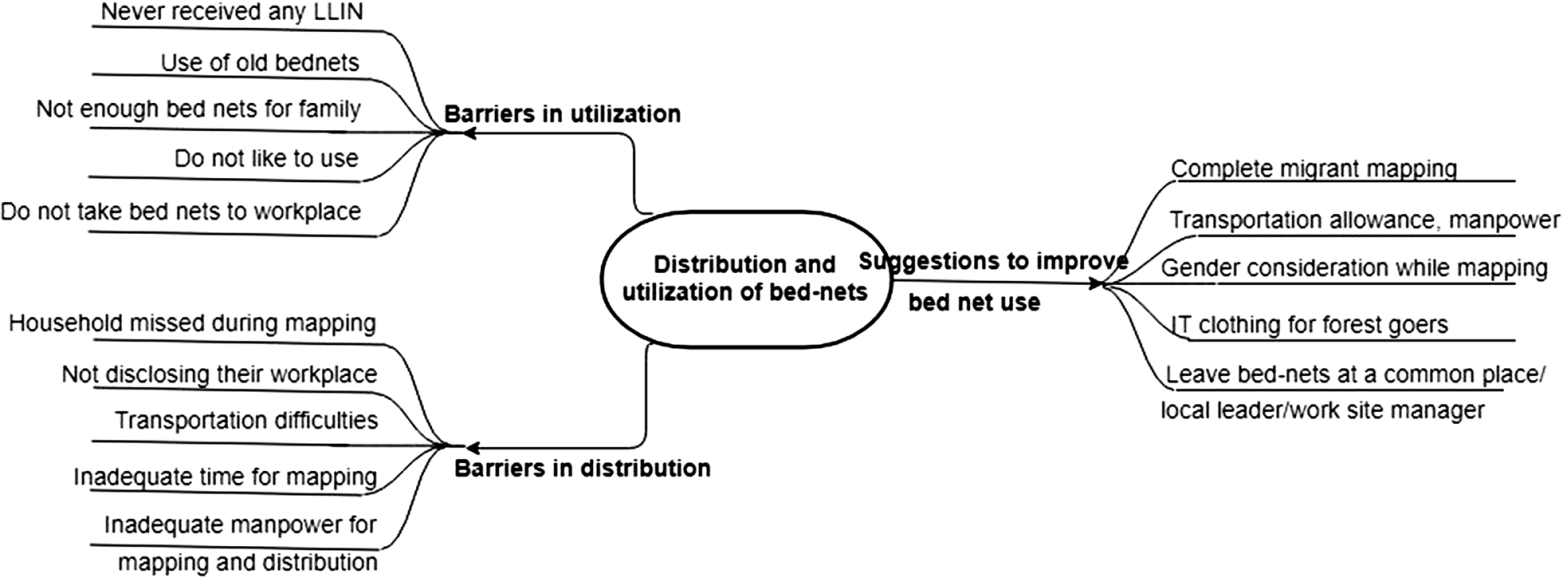
Thematic diagram showing the barriers in distribution and utilization of bed-nets among migrant population in Myanmar, 2018. *LLIN* long lasting insecticidal net, *IT* insecticide treated

The audiotapes of KIIs and FGDs were manually transcribed in Burmese language soon after data collection. This qualitative analysis employed a thematic analysis approach [20]. Thematic analysis is a method for identifying, analysing and reporting patterns (themes) within data. A theme captures something important about the data in relation to the research question, and represents some level of patterned/repeated response or meaning within the data set. A hierarchical codebook was developed by two study investigators (SYL and TMM) by synthesizing codes emerging directly from the transcripts (inductive) and from the topic guides (deductive). Both of them independently did the initial coding after going through all the transcripts (of both FGDs and KIIs) without any particular sequence as both FGDs and KIIs were related to the same research question. Then they met to discuss the codes and resolve the discrepancies. Both the coders were Masters in Public Health with training in qualitative research and a 2-week training on mixed methods research only. Atlas.ti version 7.5.4 (ATLAS.ti Scientific Software Development, Berlin, Germany) was used to code all transcripts in local language. Similar codes were then combined to generate themes. Around the global theme ‘Barriers to distribution and utilization of bednets’, three major themes emerged: (1) barriers to utilization of bednets; (2) barriers to distribution of bednets; and, (3) suggestions to improve bed-net use (Fig. 2). These themes have been adequately described in the Results section, no other minor themes were identified. Verbatims related to the themes were translated into English and presented. The study findings have been presented using the Consolidated Criteria for Reporting Qualitative Research (COREQ) guidelines [21].

## Results

### Household demographic characteristics

Table 1 shows the characteristics of migrant households in RAI areas of Myanmar. Of the 3230 migrant households surveyed, respondents from all households completed the interview (response rate of 100%). Most of them were from Tanintharyi region (27%) and Mon state (26%) followed by Sagaing region (18.3%) and Kayin (12.4%). Nearly 85% of them had no formal education or educated up to primary school level. More than one-third (38%) were either gardeners/farmers/rubber tappers, followed by workers in brick kiln/stone mining company (25%) and daily wage labourers (17.7%). Most of them have migrated to work or in search of jobs (82%).

Table 2 shows the demographic and occupational characteristics of participants of FGDs and KIIs. A total of 17 FGDs (involving 121 participants) with different group of migrants and 17 KIIs with different programme stakeholders were conducted in four selected townships.

**Table 2:** Socio-demographic details of the participants who were part of focus group discussions and key informant interviews in four townships of Myanmar 2017

FGD participants			KII participants		
Characteristics	N	%	Characteristics	N	%
Total	121	100		17	100
Gender			Gender		
Male	77	(64)	Male	14	(82)
Female	44	(36)	Female	3	(18)
Age group			Age group		
15–24 years	26	(21)	15–24 years	1	(6)
25–44 years	67	(55)	25–44 years	9	(53)
45–64 years	27	(22)	45–64 years	6	(35)
65 years and above	1	(1)	65 years and above	1	(6)
Type of participants			Type of participants		
Bamboo cutters	15	(12)	Health assistant	2	(12)
Road construction workers	15	(12)	Malaria assistant	2	(18)
Charcoal makers	11	(9)	Malaria supervisor	3	(12)
Fisherman	13	(10)	Public health supervisor	2	(12)
Gold miners	16	(13)	Village health volunteers	8	(47)
Oil diggers	19	(15)			
Stone mine workers	12	(9)			
Forest workers	3	(24)			
Teak plantation workers	6	(5)			
Others	11	(9)			
Years of service			Years of service		
0–5 years			0–5 years	8	(47)
6–10 years			6–10 years	2	(12)
More than 10 years			More than 10 years	5	(29)
Missing			Missing	2	(12)

The major themes that emerged from the KIIs and FGDs were (a) barriers in ITN utilization; (b) barriers in ITN distribution; and, (c) suggestions to improve bed net ownership and utilization (Fig. 2).

### Household ownership of and access to bed nets

Table 3 shows the household ownership of and access to bed nets among migrants in RAI areas. While almost all households, i.e., 97.8% (95% CI 97.3–98.3%) had at least one bed net, only 63.3% (95% CI 61.5–65.1) had at least one ITN. Similarly, only about 36% (95% CI 34.2–37.8%) of households had sufficient ITNs (at least one ITN per two persons in the household). About half of all household members had access to ITNs. Multivariable analysis showed that households with fewer members (< 6) had higher odds of having sufficient bed-nets. Table 4 shows qualitative analysis revealing that this was probably due to inaccurate mapping as described below. The reasons for poor ownership and access to ITNs have been linked to barriers in ITN distribution in the qualitative interviews.

**Table 3:** Household ownership and utilization of bed nets among migrant population in Regional Artemisinin-resistance Initiative areas of Myanmar, 2016

Characteristics	%	(95% CI)
Total number of households	3230	
Household ownership of bed nets		
At least one net per household (any type)	97.8	(97.3–98.3)
At least one ITN per household	63.3	(61.5–65.1)
One net per two people (any type)	69.6	(67.8–71.4)
One net per two people (ITN)	36.0	(34.2–37.8)
Total number of household members slept here last night	11,193	
Access and Utilization of bed nets		
Access to ITN	50.1	(49.2–51.2)
Reported sleeping under an ITN the previous night	52.1	(51.1–53.1)
Reported sleeping under an ITN the previous night among pregnant women	52.8	(51.0–54.4)
Reported sleeping under an ITN the previous night among under-five children	50.8	(49.0–52.8)

*ITN* insecticide treated net, *CI*  confidence interval

Figures presented here are weighted estimates; Figures given in parentheses are percentages with 95% confidence interval

**Table 4:** Socio-demographic characteristics associated with household ownership of sufficient ITNs among migrant population in the Regional Artemisinin-resistance Initiative areas of Myanmar, 2016

Characteristics	Household ownership of sufficient ITN				Unadjusted OR (95% CI)	*p* value	Adjusted OR (95% CI)	p-value
	Total	N	%	(95% CI)				
Migrant settlement type settlement^a^								
Category 3	1251	501	40.1	(37.1–43.2)	1		1	
Category 1	756	299	38.5	(34.8–42.2)	1.14 (0.57–2.29)	0.690	1.47 (0.76–2.83)	0.234
Category 2	982	334	35.0	(31.8–38.3)	1.18 (0.54–2.58)	0.658	1.07 (0.59–1.96)	0.804
Education of head of household								
Up to secondary level	197	387	36.4	(33.1–39.7)	1		1	
No formal education	260	89	33.8	(22.7–40.4)	1.28 (0.59–2.79)	0.512	1.38 (0.59–3.25)	0.434
Read and write/Primary	2464	538	34.1	(31.6–36.8)	1.37 (0.8–2.35)	0.237	1.28 (0.76–2.18)	0.332
High school and above	306	150	46.2	(40.1–52.4)	1.91 (0.97–3.77)	0.06	1.94 (0.8–4.7)	0.135
Occupation of head of household								
Daily wage labourer	572	175	28.7	(24.9–32.8)	1		1	
Farming/gardening/rubber tapper	1231	500	43.6	(40.4–46.7)	1.55 (0.78–3.08)	0.199	1.26 (0.66–2.38)	0.462
Stone or gold mining work/Oil digger/Brick kiln work	817	236	30.2	(26.8–33.8)	1.21 (0.59–2.47)	0.591	0.95 (0.52–1.73)	0.862
Merchant	38	28	43.6	(29.3–59.1)	1.24 (0.61–2.51)	0.529	1.08 (0.51–2.3)	0.824
State/region								
Kayin	401	41	10.9	(7.6–15.4)	1		1	
Tanintharyi	876	164	21.9	(19.1–25.1)	4.81 (1.64–14.1)	0.006	4.97 (1.79–13.82)	0.004
Shan (East)	27	2	1.5	(0.4–6.0)	0.18 (0.08–0.38)	< 0.001	0.07 (0.05–0.11)	< 0.001
Mon	839	455	52.0	(48.6–55.3)	12.65 (5.25–30.5)	< 0.001	11.76 (7.18–19.27)	< 0.001
Kachin	149	86	34.8	(29.1–41.0)	6.12 (2.84–13.23)	< 0.001	9.95 (5.77–17.17)	< 0.001
Sagaing	590	241	38.6	(34.1–43.3)	4.74 (1.67–13.47)	0.006	5.72 (2.56–12.78)	< 0.001
Bago	347	139	60.8	(54.3–67.0)	17.7 (8.16–38.44)	< 0.001	24.58 (13.19–45.78)	< 0.001
Number of household members								
More than 6	454	15	6.4	(3.6–11.1)	1		1	
1–2	1153	628	56.3	(52.9–59.6)	5.75 (2.73–12.12)	< 0.001	8.07 (3.87–16.79)	< 0.001
3–5	1623	508	27.0	(24.8–29.3)	1.56 (0.88–2.77)	0.12	1.51 (0.89–2.55)	0.116

Figures presented here are weighted estimates; figures given in parentheses are percentages with 95% confidence interval

*ITN* insecticide treated net, *CI* confidence interval

^a^Types of migrant settlements: Category 1 = Company owned large compound; Category 2 = Medium sized compound; Category 3 = (Owned small business-gold mining/forest workers)

### Missed populations during migrant mapping

FGDs with the migrant workers revealed that some migrants who are constantly moving, such as road workers and those working in hard-to-reach areas, are often missed during the mapping exercise and thus are excluded from the list of those eligible for bed-net distribution. Safety and security was also cited as a major concern as most of them work in difficult terrains, forest covered areas and in conflict-affected zones.

### Unwillingness to disclose their work site

KIIs with health staff found that some migrant workers, such as wood cutters, did not want to disclose their nature of work and workplace in order to avoid any legal confrontations. They would eventually be missed during the migrant mapping exercise.

### Difficulties in transportation

Transportation was the major barrier not only for migrant mapping but also for ITN distribution as the migrant sites are located in remote inaccessible areas, as some of the health staff said,

*“Reaching the place is very difficult, sometimes only by walking, need to carry nets and other things on the back. Even motorcycle won’t go that far”* (KII: Male, Public Health Supervisor).

*“I used my bullock cart for transportation. Some areas cannot be accessed by bullock cart, we have to use an elephant*”(KII: Male, Village Health Volunteer).

### Inadequate time, manpower and insufficient travel cost to conduct migrant mapping

In KII sessions, basic health staff reported that the time for migrant mapping is inadequate to catch all migrants because they live in remote locations and are often mobile due to the nature of their work. A malaria assistant said,

*“We need minimum 6* *days for area mapping. For more precise data in migrant communities, even 6* *days in not sufficient”* (KII: Male, Malaria Assistant).

Some of the KII respondents said that more manpower was required in both migrant mapping and ITN distribution. More micro planning is required in terms of the type of health worker to be employed in different areas considering the terrain, language, mode of transportation to reach the place etc.

*“For riverine route, we do not want women as volunteers as the route is dangerous. Some areas need women volunteers, Karen villages need Karen speaking workers to tackle language barrier”* (FGD: Male, Village Health Volunteer).

Cost was also a barrier in moving to these locations for migrant mapping and net distribution.

*“Some areas can only be reached* via *riverine route using a boat which costs a lot, around 80 000 kyats per boat”* (KII:Male, Malaria Assistant).

### Utilization of bed nets

About 52.1% (95% CI 51.1–53.1) reported sleeping under ITNs during the previous night. A similar proportion of pregnant women and children also reported sleeping under ITN the previous night. The reasons for low utilization of bed nets have been explored through qualitative enquiry. Most of the respondents stated that they used ITN if ITNs were available. The main barrier in ITN utilization was insufficient or no ITN in their family. The other reasons were not carrying ITNs to the work site due to overload and dislike of ITNs.

### Never received any ITNs

Most of the migrant communities got the distributed ITNs but a few of them didnt, especially road workers/fishermen who were constantly moving.

*“I sleep under ordinary bednets but not ITNs because I was not here when ITNs were distributed”* (FGD: Male, Fisherman).

*“We heard that the government staff deliver the nets, but we never got one, nevertheless, we had an old one that was also provided by health staff*” (FGD:Female, Road worker).

### Use of old bed nets

Some of the community members reported using old bed nets (more than 1 year old).

*“The one we are using now was received more than a year ago. I think the strength to kill the mosquitoes is gone”* (FGD: Male, Fisherman).

One of them was still using an old bed net despite having a new one.

*“We got two nets within a year, by different groups. The one we are using is one year old which we received first, we keep the new one for the guests”* (FGD: Male, Oil digger).

### Insufficient ITN for the family

Some migrant households have one ITN for 2 persons, but some family didnot get sufficient numbers of ITNs that they needed. In some households it was difficult to use ITNs for all despite having sufficient nets because of adult males and females in their families of different age groups.

*“I am alone and got one net, my son’s family has 7 members but he got only one net which was not sufficient for them”* (FGD:Female, Road worker).

### Not taking bed nets to their workplace

Most migrants were found not to carry bed nets with them to their workplace, especially the forest-goers or those who work away from home. The reasons cited were: (i) they did not have enough ITN at home; and, (ii) they had so many things to carry.

### Do not like to use ITNs

Some FGD respondents reported the reasons for not using bed nets were feeling hot inside the bed net, intolerance to the smell and burning sensation or allergic reaction.

*“Bad thing is burning sensation on the contact area, especially face, hot like chilli or as if bitten by ants”*(FGD: Male, Fisherman).

*“I use ordinary nets because I can’t bear the smell”*(FGD: Male, Gold miner).

### Characteristics of bed nets, including their physical condition

Table 5 shows the characteristics of surveyed bed nets at the household level. Just over half of all bed nets (54%) were ITNs. Nearly 54% of all bednets were of duration ≥ 5 years. Most of the bed nets were of one and half person size (60.6%). The main source of bed nets was government (91%). Of all nets, 32% had holes or had already undergone repairs. Regarding washing behaviour, nearly 12% of all nets were never washed, whereas another 10% are washed once or less than once a year. Most of the respondents (59.3%) reported drying the bednet under the sun.

**Table 5:** Physical condition and washing of bed nets in households among migrant population in Regional Artemisinin-resistance Initiative areas of Myanmar, 2016

Characteristics	N	%	(95% CI)
Total number bed nets	6088		
Bed net size			
One person size	429	7.0	(6.3–7.8)
One and half person size	3695	60.6	(59.3–62.0)
Two persons size	1951	32.0	(30.7–33.3)
Family size	2	0.0	(0.0–0.1)
Bed net condition			
Good (No holes)	4140	68.0	(66.7–69.1)
Repaired (No holes)	1303	21.4	(20.2–22.5)
Small holes	645	10.6	(9.5–11.5)
Type of nets			
Cotton	279	4.6	(4.0–5.2)
Nylon	361	5.9	(5.2–6.6)
Lace	1265	20.8	(19.6–21.9)
CYC	835	13.7	(12.7–14.7)
Military net	41	0.7	(0.4–0.9)
ITN	3286	53.9	(52.5–55.3)
Don’t know	21	0.4	(0.2–0.5)
Ever been soaked in insecticide			
Yes	161.9	2.7	(2.2–3.1)
Frequency of washing			
Never washed	735	12.4	(11.5–13.3)
Weekly once	187	3.2	(2.6–3.7)
Once in 2–3 weeks	577	9.8	(8.9–10.6)
Once a month	1290	21.8	(20.6–23.1)
Once in 2–3 months	1430	24.2	(23.0–25.4)
Twice a year	1110	18.8	(17.7–19.9)
Once a year	530	9.0	(8.1–9.8)
Less than once a year	50	0.9	(0.6–1.1)
Washing behaviour (N = 5349)			
Material used in bed net washing			
Soap	738	13.8	(12.8–14.8)
Soap powder/liquid/cream	4250	79.5	(78.2–80.7)
Missing	361	6.8	(5.9–7.6)
Ways of drying bed net			
In shade	2108	39.4	(37.9–40.9)
In sun	3174	59.3	(57.8–60.8)
Not sure	57	1.06	(0.7–1.3)
Washing technique			
Hand	4345	81.2	(80.0–82.5)
Foot	111	2.1	(1.6–2.5)
Stick	521	9.7	(8.9–10.6)
Missing	372	7.0	(6.1–7.8)
Source of bed net			
Gift	55	1.8	(1.2–2.4)
Government	2804	91.0	(90.0–92.1)
NGO	179	5.8	(5.0–6.7)
Pharmacy/market	17	0.5	(0.2–0.8)
Others	3	0.1	(0.0–0.1)
Don’t know	23	0.8	(0.5–1.0)
Duration of bed net			
Less than 6 months	279	4.5	(3.9–5.1)
6 months–1 year	361	5.9	(5.2–6.5)
1–2 years	1265	20.8	(19.5–21.9)
2–3 years	835	13.7	(12.7–14.7)
3–5 years	41	0.7	(0.4–0.9)
≥5 years	3286	53.9	(52.5–55.3)
Don’t know	21	0.3	(0.2–0.5)
Missing	5	0.1	(0.0–0.1)

*ITN* insecticide treated net, Weighted estimates are presented here; *CYC* Cotton 2-ply, *NGO* Non-Governmental Organization

### Suggestions to improve bed net utilization

The qualitative study also explored feasible solutions to improve bed net distribution and utilization: 1) NMCP staff suggested more time, manpower and money (transportation costs) for precise migrant mapping and effective distribution of ITNs; 2) migrants who travel to hard-to-reach areas for work suggested to leave the bed nets at a nearby common place so that they can get it later; 3) migrants also requested to give ITNs to their local group leader or work site manager when they are busy with their work or not present at the time of distribution; 4) some suggested to consider gender difference especially for the reproductive age group while mapping the population because of they cannot sleep under one net together; 5) they also proposed IT clothing for more effective prevention method of mosquito bites especially at the time of working in forests.

## Discussion

This mixed methods study among the migrants in RAI areas of Myanmar had some interesting findings. Firstly, about two-thirds of all households had at least one ITN, which is similar to another study among migrants in Bago region of Myanmar in 2014 [22]. Another study among migrant plantation workers in two regions of Myanmar showed that more than nearly 80% of households had at least one ITN [7]. This was probably because free and mass distributions of ITNs were done in both the study sites as one of the activities of the MARC Programme. This study also showed that only one-third of the migrant households had sufficient ITNs (1 ITN/2 persons) which is similar to that of another nationwide community-based study conducted in MARC areas in 2014 with 30% [23]. This is far below the desired target of 100%, suggesting the need for innovative models of ITN distribution suited to such mobile populations, some of which have been suggested by the migrants themselves in this study.

The qualitative component of this study explored barriers to distribution of ITNs, which possibly explains poor ownership of bed nets. One of them was incomplete migrant mapping due to remote locations of migrant sites, inadequate time, costs of transportation and non-availability of migrants during household visits, etc. To improve ITN distribution, accurate and detailed migrant mapping is essential because it produces a master list of migrant households eligible for ITNs. This requires more programmatic resources such as time, manpower and costs to cover transportation to reach these migrant sites.

It is reported in this study that the migrant workers were sometimes not present in the household during migrant mapping or ITN distribution due to their mobile nature of work. To tackle this, the migrants in an FGD have suggested leaving the bed nets at a nearby common place or to giving it to their local group leader or work site manager when they were not present at the time of distribution.

Secondly, only about half of all household members reported sleeping under an ITNthe previous night similar to other studies which reported a proportion ranging from 39 to 56% [22, 24, 25]. The qualitative component in this mixed methods study has tried to explore the reasons for this gap. Poor utilization of bed nets has among those who had bed nets has been ascribed to dislike of ITNs due to intolerance to the smell, allergic reaction and feeling hot inside the bed net [26]. This requires behaviour change communication (BCC) strategies through effective health messaging explaining about ITN, misconceptions around it, their minor side effects and its temporary nature. Due to the mobile and hard-to-reach nature of this population, BCC strategies could be tailor-made. A report by the WHO on BCC strategies among mobile migrant populations showed that engagement of the employers and the community is crucial in maximizing the reach of BCC programmes. It is also important to develop culturally appropriate bilingual Information Education Communication (IEC) materials [27]. BCC campaigns could be integrated with the ITN mapping and distribution activity. These messages could be reinforced during home visits by the VHVs.

BCC campaigns have been found to be effective in closing the gap between ownership and use [28, 29]. A Cochrane review on strategies to improve bed net use found that educational interventions regarding ITN use may increase the number of adults and children using them. The review also found that incentives lead to little or no difference in ownership or use of ITNs [30].

Thirdly, the qualitative enquiry reported some innovative ways of distribution of ITNs especially in such migrant/mobile populations when nobody is found at home. These may be considered by the national programme in subsequent ITN distribution campaigns. Insecticide Treated (IT) clothing was also suggested for avoiding mosquito bites for those who work in the forests. Similarly, other interventions such as insecticide-treated plastic sheeting for constructing temporary shelters, insecticide impregnated tents, insecticide-treated hammock nets, long-lasting impregnated blankets, and top sheets have been tried among refugee settlements, internally displaced populations and other complex emergencies with good success. These needs to be explored further in this setting [31].

Finally, around half of those at particular risk of malaria (children under five years and pregnant women) reported having slept under an ITN the previous night. There is no targeted strategy to distribute ITNs to these high-risk groups. A major reason is that during the migrant household mapping, the households with children and pregnant women are not listed separately. The overall target is to distribute one ITN per two people regardless of the high-risk group. This is of particular concern as malaria-related morbidity and mortality is the highest in these groups. The NMCP needs to prioritize these sub-groups to ensure they receive 100% access to ITNs.

The major strengths of the study were that the data were obtained from a large survey among migrant population in RAI areas; the response rate was high; the interviewers were well trained and supervised. Data quality was ensured through double data entry and validation using EpiData entry software and a weighted analysis was carried out to account for the multi-stage sampling design in the survey. Secondly, a mixed methods approach enabled an understanding of the challenges in distribution and utilization of bed nets in this high-risk community and also come up with feasible solutions to mitigate these challenges, both from a users and providers. Thirdly, the study also adhered to STrengthening the Reporting of OBservational Studies in Epidemiology [32] and COREQ guidelines to report the study findings [21]. Fourthly, because the survey analysed a representative sample of migrants, the results could be generalizable to this high-risk group.

A limitation of the study was that the study represents migrant population covered by migrant mapping, however, there is no information on bednet ownership and use in other hard-to-reach areas where migrant mapping could not be performed. The populations in those areas might be having poorer bed net ownership and utilization due to inaccessible locations. Another limitation might be due to the fact that the interviewers were from the malaria control programme, and healthcare workers may have been reluctant to criticize the programme.

## Conclusions

This study highlights that poor ownership and utilization of ITNs among the migrants in RAI areas of Myanmar. To achieve universal coverage and utilization, more programmatic support by NMCP is needed to carry out complete migrant mapping and continuously distribute ITNs to remote locations.

## Data Availability

Data are not available in public domain because they are currently being analyzed in related papers. However, data are available with the corresponding author (SYL).

## Annexure 1: Focus Group Discussion Topic Guide

Name of the person conducting the FGD:

- 1. Welcome
- Introduce yourself and the note taker, and send the sheet with a few quick demographic questions (age, gender, years of service) around to the group while you are introducing the focus group.

*Start with the following:*

- Who we are and what we’re trying to do
- What will be done with this information
- Why we asked you to participate

*Some ground rules*

- Everyone should participate.
- Information provided in the focus group must be kept confidential
- Stay with the group and please don’t have side conversations
- Turn off cell phones if possible
- Relax and have fun

*Topics and probes*

General Questions

- What kind of illness/diseases are commonly found in this area?
- Does malaria commonly found in this area? Which type of populations are commonly face with malaria problem?

Theme 1 knowledge about malaria, its prevention

- Do you all hear about Malaria? Other local terminology for malaria in this area? How do people get malaria? In which months of the year are people most likely to get malaria? It is preventable or not? How to prevent and why?
- What kind of preventable measure do you know
- What kinds of things usually do to protect from malaria?  Probe for the following: Mosquito nets, types of nets (treated/untreated)? Repellents, boots, clothing, smoke coils, teas..?
- Do you heard about ITNs? From whom and when? How?

Theme 2 Ownership and utilization

- Do you have bed net? Types of bed net? Is it insecticide treated or not? Numbers of bed net per household? Reason for not having sufficient bed net?
- Who provides?  When and where obtained?
- What are the most preferred products? What kind are the most popular? Why? Probe: Colour? Brand? Texture? Fabric/material? Size? Hole size?
- Sleeping habits?
- Do you use bed net? Type of bednet? Which time do you usually use bed net? Why and how? If not why? Use of ITN at home and work site?
- Do you wash net? How frequently do you wash? How and Why?
- What types of soap or detergent do you usually use? How and Why?
- How do you dry it? Why?

Theme 3 Barriers in utilization

- Are nets freely distributed or not? Why?
- Who and what organization distributed?
- Are nets distributed equally or are some people prioritized? Why?
- Are there any challenges to obtained freely distributed bed net? Why? (hard to reach, nature of work……)
- Why might some people buy nets rather than use the bed nets that are freely distributed?
- If people do not use bed nets, what do they use instead and why?
- What do people do when they sleep outside in the forest or farm to protect them from malaria? (Probe for hammock nets, repellents and other traditional methods etc.)
- Reasons for not using ITNs
  1. Occupational factors: nature of work, place of work, working hours
  2. Personal factors: knowledge, belief, attitude, habits
  3. Socio-economic factors: income, education, family size
  4. Factors related to the use of bed net
- Is there any other purpose or using bed net? (fishing, using in farming…….)

Is there any other over all suggestion?

That concludes our focus group. Thank you so much for coming and sharing your thoughts and opinions with us.

### In-depth Interview Topic Guide

Name of the person conducting the IDI:

- 2. Welcome
- Introduce yourself and the note taker, and send the sheet with a few quick demographic questions (age, gender, years of service) around to the group while you are introducing the focus group.

*Start with the following:*

- Who we are and what we’re trying to do
- What will be done with this information
- Why we asked you to participate

*Some ground rules*

- Everyone should participate.
- Information provided in the in depth interview must be kept confidential
- Stay with the people and please don’t have side conversations
- Turn off cell phones if possible
- Relax and have fun

*Topics and probes*

For providers’ perspectives,

General Questions

- What kind of illness/diseases are commonly found in this area?
- Does malaria commonly found in this area? Which type of populations are commonly face with malaria problem?
- Problem magnitude? Why?
- What are the main sources of information/communication about health for people in the community? (Probe: volunteers, facility staff, local media, radio, TV etc.).
- Which sources are the most effective? Why?
- Which sources of information do they trust most? Why?

Theme 1 Current practice of distribution of ITNs to migrants

- Distribution plan of NMCP? Current activities? Free or not? Any prioritization? How and why?
- Is there any other ITNs distributed organization? What? How? Any prioritization? Free or not? How long? Coverage area?
- Number of ITNs distributed to migrants in 2016 and 2017 by NMCP?
- Types of distributed ITNs ( Nylon, fabric….)
- Theme 2 Barriers to get Universal Coverage of ITN to migrants
- Universal Coverage of ITN to migrants or not? Why?
- Challenges in distribution of ITN to migrant population compare with static population? How and why?
- Which types of ITNs are most likely to get by migrants? Why?
- Why do migrants don`t take ITNs even it is free? Why? ( didn`t get information, hard to reach, …………)
- Is there any other misbelieve or cultural believe dislike to use ITNs? How and why?
- Theme 3 Suggestions to improve Universal Coverage of ITN to migrants
- Is there any other suggestions to overcome these barriers?
- Is there any policy and evaluation plan to overcome above challenges in distribution of ITNs to migrants?

Overall suggestion for activities of NMCP?

That concludes in depth interview. Thank you so much for coming and sharing your thoughts and opinions with us.

## Abbreviations

3MDG: Three Millennium Development Goals Fund
DMR: Department of Medical Research
DMS: Department of Medical Services
DOPH: Department of Public Health
FGD: Focus Group Discussion
GMS: Greater Mekong Subregion
ITN: insecticide-treated bed net
KII: key informant interview
LLIN: long-lasting insecticidal net
MARC: Myanmar Artemisinin Resistance Containment
NMCP: National Malaria Control Program
PI: principal investigator
RAI: Regional Artemisinin Initiative
SEAR: South-East Asia Region
WHO: World Health Organization
